# External Validity of Randomized Controlled Trials on Alzheimer’s Disease: The Biases of Frailty and Biological Aging

**DOI:** 10.3389/fneur.2017.00628

**Published:** 2017-11-27

**Authors:** Marco Canevelli, Alessandro Trebbastoni, Federica Quarata, Fabrizia D’Antonio, Matteo Cesari, Carlo de Lena, Giuseppe Bruno

**Affiliations:** ^1^Department of Neurology and Psychiatry, Sapienza University of Rome, Rome, Italy; ^2^Fondazione IRCCS Ca’ Granda, Ospedale Maggiore Policlinico, Milan, Italy; ^3^Geriatric Unit, Department of Clinical Sciences and Community Health, University of Milan, Milan, Italy

**Keywords:** Alzheimer’s disease, frailty, randomized controlled trial, external validity, aging, holistic approach

## Abstract

To date, the external validity of randomized controlled trials (RCTs) on Alzheimer’s disease (AD) has been assessed only considering monodimensional variables. Nevertheless, looking at isolated and single characteristics cannot guarantee a sufficient level of appreciation of the AD patients’ complexity. The only way to understand whether the two worlds (i.e., research and clinics) deal with the same type of patients is to adopt multidimensional approaches more holistically reflecting the biological age of the individual. In the present study, we compared measures of frailty/biological aging [assessed by a Frailty Index (FI)] of a sample of patients with AD resulted eligible and subsequently included in phase III RCTs compared to patients referring to the same clinical service, but not considered for inclusion. The “RCT sample” and the “real world sample” were found to be statistically similar for all the considered sociodemographic and clinical variables. Nevertheless, the “real world sample” was found to be significantly frailer compared to the “RCT sample,” as indicated by higher FI scores [0.28 (SD 0.1) vs. 0.17 (SD 0.1); *p* < 0.001, respectively]. Moreover, when assessing the relationship between FI and age, we found that the correlation was almost null in the “RCT sample” (Spearman’s *r* = 0.01; *p* = 0.98), while it was statistically significant in the “real world sample” (*r* = 0.49; *p* = 0.02). The application of too rigid designs may result in the poor representativeness of RCT samples. It may even imply the study of a condition biologically different from that observed in the “real world.” The adoption of multidimensional measures capable to capture the individual’s biological age may facilitate evaluating the external validity of clinical studies, implicitly improving the interpretation of the results and their translation in the clinical arena.

## Introduction

Randomized controlled trials (RCTs) represent the gold standard for establishing the efficacy and safety/tolerability of medical interventions. Besides providing information concerning clinical and therapeutic outcomes, RCTs should also try to generate messages that are externally valid or generalizable as much as possible. In other words, they should provide information that can be translated from the relatively small group of RCT participants to a larger scale population (1). Unfortunately, the rigid and idealized conditions typical of RCTs may often compromise the transferability of the study findings to the “real world” clinical practice, finally affecting their usefulness. In particular, if the RCT eligibility criteria are too selective (in order to maximize the focus on a specific aspect of research), the representativeness of the sampled population and the following generalizability of the results will be challenged.

Alzheimer’s disease (AD) is growingly indicated as a research priority (2). To date, there are more than 140 phase II–IV studies currently exploring the efficacy of different interventions with the aim of improving the clinical manifestations of the disease and/or positively influencing its natural course (source: clinicaltrials.gov). The external validity of RCTs on AD has repeatedly been debated. Specifically, a substantial discrepancy has been shown between patients enrolled in RCTs and the patients’ population which should benefit from the studies results (3, 4). Moreover, there is a relative lack of information about the comparability of these two populations in terms of clinical and pharmacological characteristics (5). At the same time, it seems that RCTs on AD pay special attention to other aspects for selecting the populations of interest. For example, a good representativeness with regard to sex distribution has been reported for most of available RCTs (6).

It is possible that relying on specific sociodemographic factors (e.g., age and sex) and/or crude clinical parameters (e.g., number of diseases, type of comorbidities, concomitant medications) only provides a monodimensional (and not comprehensive) evaluation of the RCTs participants’ health status. Looking at isolated and single variables cannot guarantee a sufficient level of appreciation of the AD patients’ complexity. Moreover, it will preclude a fair comparability between RCTs participants and the overall population of patients with AD. The only way to understand whether the two worlds (i.e., research and clinical practice) deal with the same type of patients is to adopt a multidimensional approach relying on measures/variables that are able to more holistically reflecting the biological age of the individual.

In the present study, we assess the biological aging [measured by a Frailty Index (FI)] presented by a sample of AD patients resulted eligible and subsequently included in phase III RCTs compared to patients referring to the same clinical service, but not considered for inclusion.

## Materials and Methods

### Participants

The present analyses were conducted by retrospectively reviewing the clinical charts of patients with AD attending the Department of Neurology and Psychiatry of the “Sapienza” University of Rome (Italy) between January 2015 and January 2017.

We compared the clinical and sociodemographic characteristics of two groups of patients, identified as follows:

– The “RCTs sample” was composed by patients meeting the following inclusion criteria shared by two phase III RCTs ongoing at our department, both exploring the efficacy and safety/tolerability of passive immunization interventions against amyloid: (1) age between 55 and 90 years; (2) diagnosis of probable AD dementia [NIA-AA criteria (7)]; (3) Mini-Mental State Examination (MMSE) score ranging between 20 and 26; (4) absence of concurrent serious or unstable illnesses (investigator’s opinion); and (5) positive findings at the amyloid positron emission tomography scan or cerebrospinal fluid consistent with the presence of amyloid pathology [i.e., probable AD dementia with evidence of the AD pathophysiological process (7)].
– The “real world” sample was composed by an equal number of consecutively selected, age- and MMSE-matched patients with AD attending our memory clinic, not enrolled in any RCTs. In order to mirror as much as possible the eligibility criteria selecting the “RCTs sample,” the following inclusion criteria were additionally adopted for the “real world sample”: (1) age between 55 and 90 years; (2) diagnosis of probable AD dementia (NIA-AA criteria); (3) MMSE score ranging between 20 and 26; and (4) absence of concurrent serious or unstable illnesses.

The present study did not require formal ethical approval in accordance with the national and institutional guidelines. Patients and caregivers (or legal guardians when necessary) provided written informed consent for allowing the utilization of the collected data for research purposes (as required by the “Policlinico Umberto I” university hospital of Rome). Data used in the present analyses were exclusively retrieved from medical charts where information was recorded as part of the standard clinical routine.

### Frailty Index

A FI was retrospectively generated from the variables available in the clinical charts following a standard procedure (8) by computing 28 age-related deficits (including signs, symptoms, adjudicated diagnoses, disabilities) (Table 1). Each item included in the FI was coded so that a value of 0 indicated the absence of the deficit and a value of 1 its presence. The FI was calculated as the ratio between the number of deficits presented by the individual and the number of considered deficits (i.e., 28). Thus, the FI potentially ranged between 0 (no deficit) and 1 (all deficits).

**Table 1:** Deficits included in the computation of the 28-item Frailty Index.

1. Hypertension
2. Autoimmune disease
3. Hepatic diseases
4. Ischemic heart disease
5. History of TIA/stroke
6. Diabetes
7. Focal neurological signs
8. Renal failure
9. Arrhythmia
10. Thyroid disease
11. Cancer
12. COPD
13. Dyslipidemia
14. Obesity (BMI ≥ 30 kg/m^2^)
15. Underweight (BMI < 18.5 kg/m^2^)
16. Urinary incontinence
17. Depression
18. Apathy (NPI)
19. Anxiety (NPI)
20. Sleep disorders
21. IADL (shopping)
22. IADL (transportation)
23. IADL (drugs)
24. IADL (money)
25. ADL (toileting)
26. ADL (eating)
27. ADL (dressing)
28. ADL (transportation)

*ADL, activities of daily living; BMI, body max index; COPD, chronic obstructive pulmonary disease; IADL, instrumental activities of daily living; NPI, neuropsychiatric inventory*.

### Statistical Analyses

Univariate analyses were conducted to compare the baseline data between the “RCT sample” and the “real world sample.” Spearman’s correlations were used to assess the strength and direction of the relationship between age and FI.

## Results

**Figure 1:**
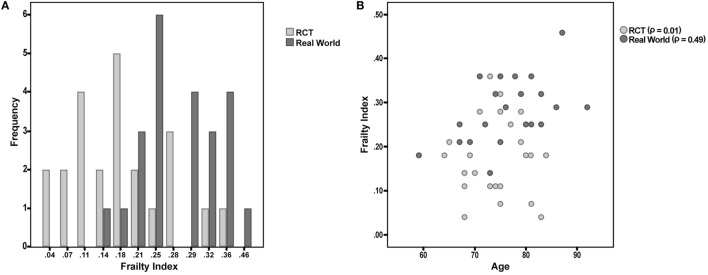
**(A)** Frailty Index (FI) values among participants enrolled in a phase III randomized controlled trial (RCT) and attending a “real world” memory clinic. **(B)** Correlation between age and FI in the two considered samples of patients.

Overall, the “RCT sample” and the “real world sample” were found to be statistically similar for all the considered sociodemographic and clinical variables (all *p* values > 0.05; Table 2). Nevertheless, the “real world sample” was found to be significantly frailer compared to the “RCT sample,” as indicated by higher FI scores [0.28 (SD 0.1) vs. 0.17 (SD 0.1); *p* < 0.001, respectively] (Figure 1A). Consistently, the majority (78.3%) of patients in the “real world sample” were frail [i.e., FI ≥0.25 (9)], whereas only 26.1% of frailty prevalence was reported in the “RCT sample” (*p* < 0.001). The statistically significant difference in FI between the two groups was confirmed even after adjustments for age and sex (data available upon request).

**Table 2:** Sociodemographic and clinical characteristics of the study samples.

	RCT (*n* = 23)	Real world (*n* = 23)	*p*
Age (years)	74.2 ± 5.7	76.6 ± 7.9	0.26
Sex (F)	52.2	56.5	0.77
Education (years)	10.1 ± 4.9	9.8 ± 5.1	0.84
BMI (kg/m^2^)	25.4 ± 3.2	24.5 ± 3.4	0.37
Familial history for AD	47.8	33.3	0.33
Hypertension	43.5	65.2	0.14
Diabetes	13.0	13.0	1.0
Dyslipidemia	52.2	52.2	1.0
COPD	8.7	13.0	0.64
Ischemic heart disease	8.7	4.3	0.55
MMSE	22.0 ± 1.6	22.3 ± 2.0	0.68
ADL	5.6 ± 0.6	5.2 ± 1.3	0.12
Drugs	4.9 ± 1.7	4.1 ± 2.1	0.17
Polypharmacy	34.8	39.1	0.76
Frailty Index	0.17 ± 0.1	0.28 ± 0.1	< 0.001

*Data are expressed as %, or mean ± SD*.

*Polypharmacy was defined as taking five or more concomitant medications*.

*AD, Alzheimer’s disease; ADL, activities of daily living; BMI, body max index; COPD, chronic obstructive pulmonary disease; MMSE, Mini-Mental State Examination; RCT, randomized controlled trial*.

Finally, the correlation between FI and age was tested in the two samples. The correlation was almost null in the “RCT sample” (Spearman’s *r* = 0.01; *p* = 0.98), while it was statistically significant in the “real world sample” (*r* = 0.49; *p* = 0.02) (Figure 1B).

## Discussion

In the present study, a marked discrepancy in terms of frailty status/biological aging was observed between patients with AD eligible for RCTs versus those evaluated in the same clinical setting but not considered for inclusion. Interestingly, only a minority of the patients selected for phase III RCTs could be considered as frail, despite the fact that these persons were all affected by an overt dementia condition (even if of mild entity). On the other hand, most of patients undergoing the standard clinical procedures exhibited a relevant amount of chronic deficits. The non-significant correlation between frailty and age in the “RCT sample” is particularly noteworthy. The absence of an age-related accumulation of deficits implicitly indicates that the studied condition is isolated from the natural aging process. It means that the AD is studied without taking into account the progressive failure of the organism reserves due to aging. It is evident as such scenario (i.e., a disease unaffected by the natural life course) is purely theoretical and of hard reproducibility in the “real world”.

These results can raise doubts about the applicability of findings coming from RCTs on AD to the “real world” clinical practice. In particular, how is it possible to transfer evidence obtained in a population of robust individuals to a population of frail subjects with multiple and interacting deficits? How and to what extent can this discrepancy affect the efficacy and safety/tolerability of the tested interventions in the routine clinical practice? Our analyses confirm how the AD population encountered in the daily practice is poorly comparable with the highly selected RCT participants. In fact, the same target condition is identified by adopting different approaches, one relying on probability criteria (i.e., “real world”) whereas the other requires a high level of characterization including the demonstration *in vivo* of specific pathophysiological modifications. Such ambiguity automatically influences the phenotypic and biological characteristics of the condition of interest in the two settings, challenging their comparability and consistency.

Our data also confirm that the adoption of monodimensional variables may be inadequate for capturing the inner characteristics of individuals affected by multiple age-related conditions. In fact, singularly looking at the collected sociodemographic and clinical data, one may suppose the similarity of our two populations, potentially leading to erroneous conclusions of adequate comparability and representativeness. Differently, the adoption of a multidimensional measure (as the FI), providing a more comprehensive estimate of the individual’s biological status, allowed to reveal substantial discrepancies between the two groups. Under this perspective, a comprehensive assessment of the (frail) older individual is necessary and may improve clinical and research standards (10). In this context, the condition of frailty has already been linked to typical pathophysiological modifications of AD (e.g., amyloid deposition). However, it is still unclear whether the demonstrated association is due to a direct (e.g., amyloid depots are cause of frailty) or indirect (e.g., amyloid accumulation is related to frailty because they are both age-related conditions) mechanism. Indeed, the existing literature is quite ambiguous at nesting the effects of the aging process in the pathophysiological mechanisms leading to AD. Thus, in our study, we used a measure of frailty (intended as biological aging) for verifying the appropriate consideration of “aging” in the AD construct used in the clinical and research setting.

Multiple instruments are available in the literature for measuring the frailty condition. We chose to rely for our study on the FI for multiple reasons. Differently from other tools, the FI provides a multidimensional assessment of the individual’s biological age. In other words, it is not focused on a specific domain or characteristic (e.g., physical function) of the health status, but provides a comprehensive weighting of the deficits burdening the organism. In this context, it is also important to mention that the FI is based on a quantitative (and not qualitative) approach to the frailty condition. This means that it can be computed relying on the retrospective use of already existing data collected for different purposes. Such unique characteristic allows its computation without the need of modifying the clinical and research practice already in place (i.e., it is not based on pre-defined questions or items) (10, 11).

Our study has some limitations. The limited sample size does not consent to draw definitive conclusions on the topic. However, the strength and consistency of the findings may suggest the absence of false positive results. We considered patients enrolled in a limited number of specific RCTs (i.e., two). Nevertheless, the eligibility criteria were similar to those considered by the majority of the ongoing RCTs in the field. The number of deficit used to compute the FI in our study (i.e., 28) was lower than what recommended in the standard procedures (i.e., 30) (8). The design of our study did not allow us to explore the contribution of specific factors (e.g., the implicit tendency of investigators to recruit healthier individuals in RCTs, the willingness of patients to participate in the RCT, etc.) potentially explaining the observed differences.

In conclusion, the application of too rigid designs in RCTs (especially those conducted in biologically old individuals) may result in the poor representativeness of participants for the “real world” clinical population they should mirror. The adoption of multidimensional measures (such as the FI) capable to capture the biological age of the person may facilitate the evaluation of the external validity of clinical studies, implicitly improving the interpretation of the results and their translation in the clinical arena.

## Ethics Statement

The present analyses were conducted only retrospectively considering data that were routinely collected in the daily clinical practice. No additional experimental procedure was performed within the study.

## Author Contributions

MCanevelli, MCesari, and GB designed the study and wrote the manuscript. AT, FQ, FD, and CL participated in data collection. All the authors contributed to the critical interpretation of the findings and to the drafting of the manuscript.

## Conflict of Interest Statement

MCesari has received honoraria for presentations at scientific meetings and/or research fundings from Nestlé and Pfizer. He is involved in the coordination of an Innovative Medicines Initiative-funded project [including partners from the European Federation Pharmaceutical Industries and Associates (Sanofi, Novartis, Servier, GSK, Lilly)]. The other authors have no conflict of interest to declare.
